# Participation and satisfaction of prostate cancer patients on androgen deprivation therapy participating in a virtual supervised group exercise service

**DOI:** 10.1002/bco2.70243

**Published:** 2026-09-30

**Authors:** Emily Curtis, Alice Russell, Chris Cottrell, Siobhan Cowan‐Dickie, Sarah Dewhurst, Netty Kinsella, Yae‐eun Suh, Declan Cahill, Louis Fox, Mieke Van Hemelrijck, Alison Reid

**Affiliations:** ^1^ The Royal Marsden NHS Foundation Trust London UK; ^2^ Transforming cancer OUtcomes through Research (TOUR), King's College London London UK; ^3^ The Exercise Clinic Ltd London UK

**Keywords:** aerobic, androgen deprivation, app, exercise, hormone therapy, prostate cancer, resistance, virtual

## Abstract

**Objectives:**

To evaluate participation, satisfaction and selected health‐related outcomes and to explore factors influencing engagement with a digitally delivered exercise programme among people with prostate cancer receiving androgen deprivation therapy (ADT) who took part in The Exercise Clinic® (TEC) component of the THRIVE service at The Royal Marsden NHS Foundation Trust.

**Patients and methods:**

THRIVE was developed at The Royal Marsden NHS Foundation Trust in collaboration with a private exercise provider, TEC, to improve access to exercise services for prostate cancer (PCa) patients. Between November 2020 and December 2023, 148 men on ADT for more than 6 months were enrolled in the TEC component of THRIVE. Participants received a home exercise programme, weekly virtual supervised group exercise sessions and access to the TEC mobile phone application (app) to record activity. Primary outcomes were participation and satisfaction. Secondary outcomes included activity levels, physical performance and quality of life. Quantitative outcomes were measured at baseline and quarterly reviews; exploratory analyses examined changes between baseline and 12 months using paired *t*‐tests. Qualitative data, collected via discussion groups and feedback questionnaires, was analysed using framework analysis to explore factors influencing engagement.

**Results:**

Participants attended 14 exercise sessions during the first 6 months. 88%, 90% and 88% of eligible participants completed their 3‐, 6‐ and 12‐month review, respectively. Almost two‐thirds of participants used TEC app. Overall experience was rated highly (median 10 out of 10). Virtual delivery and peer support were identified as key factors influencing engagement. Statistically significant changes were observed in EQ‐5D index values between baseline and 12 months and in sit‐to‐stand performance and average daily step count from quarter one to four.

**Conclusion:**

This service evaluation demonstrates high engagement and satisfaction among a selected group of men receiving long‐term ADT participating in a virtually delivered, supervised exercise programme embedded within routine care. Factors such as social support and the accessible delivery format may contribute to engagement with exercise in this population. Observed changes in patient‐reported and functional outcomes should be interpreted cautiously given potential selection bias and the lack of a comparator group. Findings support the feasibility and acceptability of digitally delivered exercise support extending beyond 3 months and provide further insight into the factors that may inform the design of future interventions. Future randomised controlled trials are needed to evaluate clinical effectiveness, cost‐effectiveness and the feasibility of wider implementation.

## 1 INTRODUCTION

The widespread use and clinical benefit of androgen deprivation therapy (ADT) for patients with prostate cancer (PCa) necessitates strategies to counter its adverse effects, including fatigue, reduced muscle and bone mass, increased fat mass and low mood.^1^, ^2^ Exercise training is an evidence‐based intervention shown to minimise and, in some cases, reverse ADT‐related toxicities.^3^, ^4^, ^5^ Despite this, engagement in physical activity (PA) remains low; in the United Kingdom, approximately 31% of individuals living with and beyond cancer are completely inactive,^6^ and many PCa survivors do not meet national PA guidelines.^7^, ^8^

Additional barriers PCa patients face, including comorbidities, treatment side effects and insufficient confidence and motivation, mean that access to targeted programmes is paramount^9^, ^10^, ^11^; specifically, exercise programmes for people with cancer should be supervised, individualised and conducted in group settings.^12^ Understanding how these factors influence engagement with exercise interventions is important for optimising programme design and delivery.

The National Institute for Health and Care Excellence (NICE) clinical guidance published in 2014 recommends people starting or undergoing ADT be offered supervised resistance and aerobic exercise twice a week for 12 weeks (NH131 1.4.19).^13^ Despite this, provision within the UK National Health Service (NHS) is scarce: a multicentre investigation identified only three such programmes across the United Kingdom,^14^ suggesting that most people with PCa are not adequately supported to meet PA guidelines.^9^, ^14^

To increase access to exercise within public funding constraints and an increasingly digital healthcare landscape, online programmes offer a potential solution, with theoretical advantages of lower costs, ease of scalability and reduced time, transport and financial burdens for patients.^15^, ^16^ When this service was commenced, there was limited evidence for supervised, group‐based exercise programmes delivered virtually. One pilot study among women receiving hormone therapy for breast cancer demonstrated feasibility and significant improvements in physical function, energy levels, emotional wellbeing and pain^16^; however, sex differences in body composition and exercise preferences mean findings are not necessarily generalisable to those with PCa.

THRIVE was established in 2020 as a collaborative pathway between oncologists and physiotherapists at The Royal Marsden NHS Foundation Trust (RM), a tertiary cancer centre, and The Exercise Clinic® (TEC), a private exercise provider. The pathway (Figure 1) involves referral, triage and risk stratification, including physiotherapy assessment for exercise safety, followed by onward referral for an education seminar and either physiotherapy support (provided by RM physiotherapy team) or a virtual group exercise programme (provided by TEC). THRIVE aimed to improve access to education and exercise services and increase activity levels among PCa patients in line with best‐practice recommendations. A service evaluation was approved with the objectives of assessing participation and satisfaction in TEC service components of the THRIVE pathway. Among those referred into THRIVE, the number of patients eligible to participate in the TEC service was assessed. Other aspects evaluated included how the service affected participants' activity levels, physical performance and quality of life (QoL). The service aimed to achieve preservation of activity, physical performance and QoL. This paper reports findings for participants who were on long‐term ADT (for more than 6 months) for PCa and were enrolled in the TEC component of THRIVE between November 2020 and December 2023.

**FIGURE 1:**
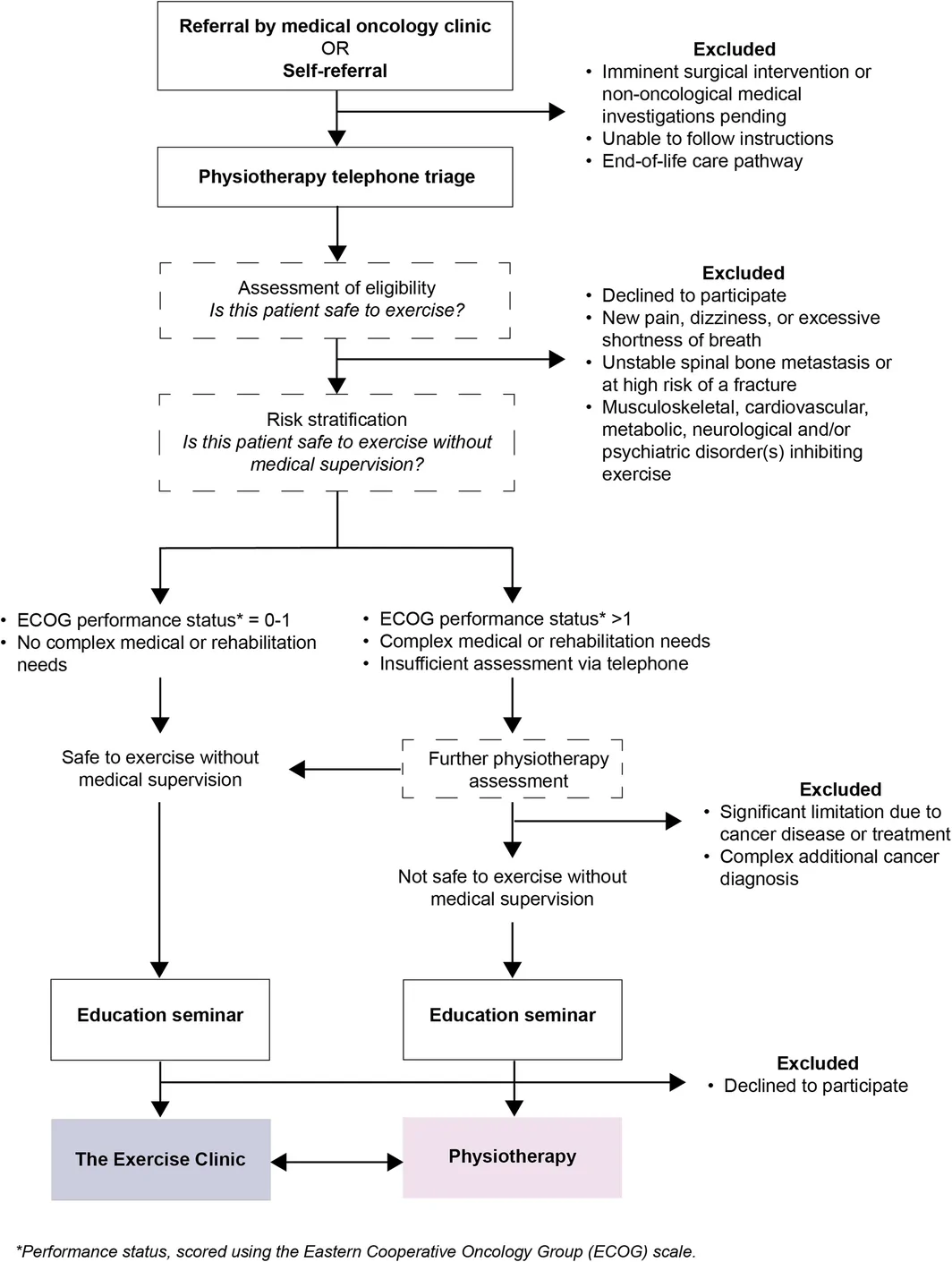
The THRIVE referral pathway and risk assessment procedure.

## 2 PATIENTS AND METHODS

This service evaluation (SE1412) was approved by RM committee as a retrospective evaluation of the TEC component of the THRIVE pathway. THRIVE was initiated in November 2020, with the initial intention of running for 12 months. This included funding for a physiotherapist (0.4 WTE). Due to additional budget availability, the TEC component was extended to December 2023.

### 2.1 Population

All patients receiving treatment for PCa at RM Sutton were eligible for referral by the oncology clinic or self‐referral to THRIVE. Patients with imminent surgical interventions or non‐oncological investigations pending, unable to follow instructions, or on an end‐of‐life care pathway were excluded. Following referral, men gave verbal consent and were assessed by a physiotherapist for exercise safety. Assessments were conducted via telephone, with an additional individual assessment (conducted in‐person or via video call) if necessary. Those with symptoms or diagnoses that prevented safe exercise (e.g. excessive shortness of breath, uncontrolled hypertension) were excluded. Those with confirmed or suspected cardiac issues were referred to their general practitioner (GP) or cardiologist for clearance to participate. Patients safe to exercise but with complex medical or rehabilitation needs (e.g. unstable spinal bone metastasis, high risk of a fracture, frailty) were offered physiotherapy. Those safe to exercise independently without medical supervision were offered an exercise programme with behavioural support provided by TEC. Continuous collaboration between RM physiotherapists and TEC allowed participants to be referred between the two pathways following initial allocation if necessary. As a result, some participants accessed physiotherapy and TEC service.

After triage and risk stratification, all potential participants were invited to an education seminar (conducted via video call) to provide them with further details about THRIVE, counselling about exercise safety and the opportunity to meet the physiotherapy and TEC teams. Participants provided written informed consent (via an online form) and completed baseline assessments (via a virtual individual review appointment and online health assessment questionnaire [HAQ]).

### 2.2 TEC

Patients triaged to TEC had access to supervised group exercise sessions, an individualised home exercise programme, activity monitoring via a mobile application (app) and quarterly individual reviews (Figure 2). The service was led by a clinical exercise physiologist (CEP) with cardiac and cancer and exercise rehabilitation training and a patient contributor (PC). Men participated until they felt confident to self‐manage exercise or withdrew.

**FIGURE 2:**
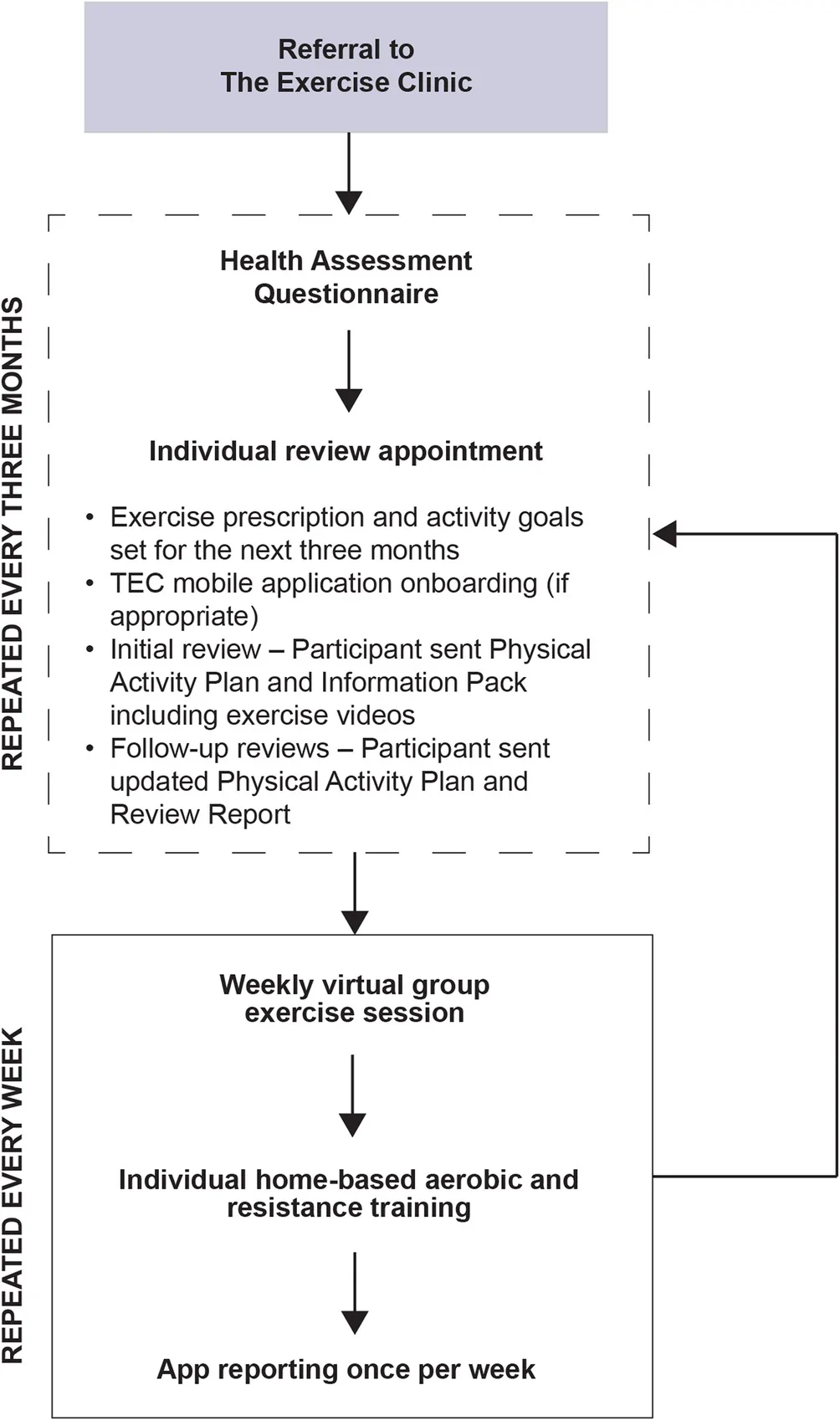
The Exercise Clinic (TEC) service and follow‐up procedure.

Group exercise sessions were led by the CEP and PC. Sessions were held once a week, conducted via video call, in groups of up to 12 participants, lasting approximately 60 min. They followed a general format of a warm‐up, mobility exercises, a series of moderate‐intensity aerobic and resistance exercises, a cool‐down and stretches. The aerobic component consisted of moderate‐impact exercises, including toe‐tap, march, heel kick and sit‐to‐stand movements. Intensity was autoregulated individually at five to seven on the 10‐point Borg rating of perceived exertion (RPE).^17^ The resistance component involved upper and lower bodyweight strength exercises, including squat, hip hinge and wall press. Sessions were tailored to individual capability and comorbidities, with low‐impact alternatives and opportunities for progression. Participants were encouraged to supplement group sessions with an individualised prescription of home‐based aerobic and resistance exercises, conducted independently but with explanatory videos available for guidance.

Behavioural support, provided at group exercise sessions, quarterly individual reviews and via TEC app, was incorporated to maximise adherence and encourage long‐term maintenance of exercise. It was informed by theory (including social cognitive theory,^18^ habit theory^19^ and the behaviour change wheel^20^) and evidence (including a Cochrane review of exercise behaviour among cancer patients^9^). Behavioural change techniques of setting programme and individual goals, self‐monitoring of behaviour and prompting of practice were employed. During exercise sessions, the CEP provided ongoing feedback and intensity guidance. At individual reviews, conducted every 3 months via video call, the CEP and PC (both trained in PA counselling) helped participants identify barriers to exercise and encouraged self‐management strategies. Self‐reported activity data and outcome measurements from the previous quarter were reviewed, informing an individualised exercise prescription and activity goals (steps per day, aerobic sessions per week, strength sessions per week) for the next quarter. This aimed to support men to achieve the national recommendations for PA of at least 150 min of moderate‐intensity or 75 min of vigorous‐intensity exercise and two strength sessions per week.^8^

TEC app aimed to encourage activity levels and accountability. Users were asked to submit their average daily step count, total number of aerobic sessions, total number of strength sessions and a rating of their physical wellbeing, emotional wellbeing, diet and sleep quality each week. For participants who declined or were unable to use the TEC app, data were recorded in a Physical Activity Plan and step count tracked using a pedometer.

### 2.3 Modifications to the programme

THRIVE was iteratively refined throughout its existence based on uptake, engagement and participant feedback. The education seminar proved inconvenient for participants due to work, social or healthcare commitments and was replaced with a one‐to‐one phone call with the PC. Group exercise sessions were initially scheduled once every 3 months. In response to participant availability, positive feedback and lower than anticipated patient referrals, this was changed to once‐weekly sessions. Assessment by RM physiotherapy for exercise safety identified several potential participants with exercise limitations. To maximise the opportunity for supervised exercise with TEC, an exclusively seated group exercise session was offered to those with limited mobility, balance or strength, conducted in a smaller group of up to six participants, lasting approximately 30 min.

### 2.4 TEC outcome measures

Primary outcomes were engagement and participant satisfaction. Secondary outcomes included changes to activity levels, physical performance and QoL. Feasibility and safety were explored with particular focus on the triage process and communication between oncology, physiotherapy and TEC teams.

#### 2.4.1 Engagement

The number of patients eligible to participate in TEC and the number who agreed to participate were recorded. To assess engagement, attendance at group exercise sessions and individual reviews and use of TEC app (number of users, reasons for digital exclusion, and number of reports submitted) were recorded weekly. A participant was defined as engaged in TEC if they had participated in their most recent quarterly review and had attended an exercise session or submitted a TEC app report in the previous month. As participants were recruited sequentially throughout the service period, the percentage of eligible participants who attended their individual review at each follow‐up point was recorded (i.e. of those who had participated in TEC for 6 months, what percentage attended their 6‐month review). Adverse event occurrence was also recorded during individual reviews. Results from similar interventions^21^, ^22^ were used to define targets of 75% and 60% attendance at 6‐ and 12‐month review, 70% attendance at group exercise sessions during the first 6 months, and 50% of participants using TEC app.

#### 2.4.2 Participant satisfaction

Overall experience of TEC service was assessed on a scale of 0 (*worst*) to 10 (*best*) via a feedback questionnaire approved by RM and sent to all eligible participants in February 2022 (some participants had not yet been referred to the programme and were therefore not able to complete the questionnaire). An average rating of 7 out of 10 was set as a target. After the service, participants were invited to take part in patient discussion groups. During these discussion groups, TEC asked participants questions about their experience including what led them to take part in the service and what their overall view of the service was.

#### 2.4.3 Activity, physical performance and QoL

The Godin–Shephard leisure–time PA questionnaire^23^ was used to assess activity levels. Godin–Shephard score was calculated using data collected in the HAQ, completed at baseline and prior to each quarterly review. The HAQ was adapted from previous survey tools used in cancer populations.^24^, ^25^ Godin–Shephard score was supplemented by step count, collected via TEC app or using a pedometer and reported as an average over each 3‐month period. Physical performance was assessed using the sit‐to‐stand test, carried out quarterly during group exercise sessions. This test of lower body functional capacity was chosen for its clinical relevance to this patient population and feasibility (minimal space and equipment requirements). QoL was measured using the EQ‐5D questionnaire^26^ completed at baseline and each quarterly review.

#### 2.4.4 Secondary outcomes

Other secondary outcomes were collected as part of TEC's routine data capture and included information collected via TEC app or HAQ. This information was used to inform quarterly reviews and individualised exercise prescriptions. Outcomes were patient‐reported changes in physical and emotional wellbeing, body mass index (BMI) and waist circumference. Physical and emotional wellbeing scores, assessed on a scale of one (*lowest*) to 10 (*highest*), were collected each week via TEC app and used to calculate an average score for each quarter. BMI, derived from height and weight measurements, and waist circumference were measured by participants and recorded in the HAQ. Additionally, subjective effectiveness of the intervention on balance, walking, strength, pain, fatigue and activity levels was assessed via the feedback questionnaire. Additional demographic and clinical data were collected at baseline.

### 2.5 Statistical analyses

Quantitative data were analysed using STATA version 18. Descriptive statistics were used to characterise the participant population, presented as mean [standard deviation (SD)] or *n* (%). Summary statistics for outcome variables at each assessment time point were reported, presented as mean (SD), median [interquartile range (IQR)] and mean difference [95% confidence interval (CI)]. Changes in outcome measures from baseline to 12 months were assessed for statistical significance using paired *t*‐tests. As sit‐to‐stand and step count were not assessed at baseline, changes in these outcomes were assessed between 3 and 12 months. All tests used a complete case approach and were two sided, with statistical significance set at an alpha level of 0.05.

Qualitative data were analysed using framework analysis,^27^ with NVivo (version 12) for data management. Participant comments were read and re‐read to achieve data familiarisation. The first three comments were triple coded by A.R., C.C. and E.C., who individually derived codes and then collaboratively developed an analytical framework. The initial framework was then applied to two further comments for refinement. The finalised framework was used by A.R. to independently code the remaining comments. Codes were arranged into categories using a matrix. Key components were identified by comparing within and between categories and further refined and defined through discussion between the research team. Participants provided written consent for the use of anonymised quotes used in this paper.

## 3 RESULTS

### 3.1 Quantitative

#### 3.1.1 Engagement

Patients due to receive or receiving treatment for PCa were introduced to the THRIVE pathway from November 2020 to December 2023. Of those that completed the physiotherapy triage, 45 were not suitable for TEC remote‐led service and were supported solely by the physiotherapy team. The remaining 221 men were invited to participate in the TEC programme, either following physiotherapy triage or after direct referral when no funded physiotherapist was in post. Twenty‐eight of these patients had received physiotherapy prior to referral to TEC.

After the education seminar and baseline assessments, 53 men (24%) were excluded. Eleven were placed on hold pending medical clearance to exercise, and four were re‐referred to physiotherapy for specialist one‐to‐one support. Five were unable to participate due to digital inaccessibility; these participants did not own a computer or smart phone or were not comfortable with using the video platform to access group exercise sessions. Ten were unable to be contacted or were initially contacted, and then contact was lost. Ten were unable to participate due to work commitments. Twelve declined to participate; reasons for declining to participate comprised content with their own exercise routine (*n* = 4), busy (*n* = 3), not interested (*n* = 2), side effects from treatment (*n* = 1), an additional health concern (*n* = 1) or no reason (*n* = 1). One patient requested to use TEC app to report his weekly exercise but did not want to participate in other components of the programme.

The remaining 168 patients provided written informed consent to participate and were enrolled in TEC programme (Figure 3). Of these 168 participants, 148 were currently on or due to receive ADT for 6 months or longer. Among the 148 participants, 91 (61%) were receiving ADT in conjunction with an androgenic receptor pathway inhibitor (ARPI).

**FIGURE 3:**
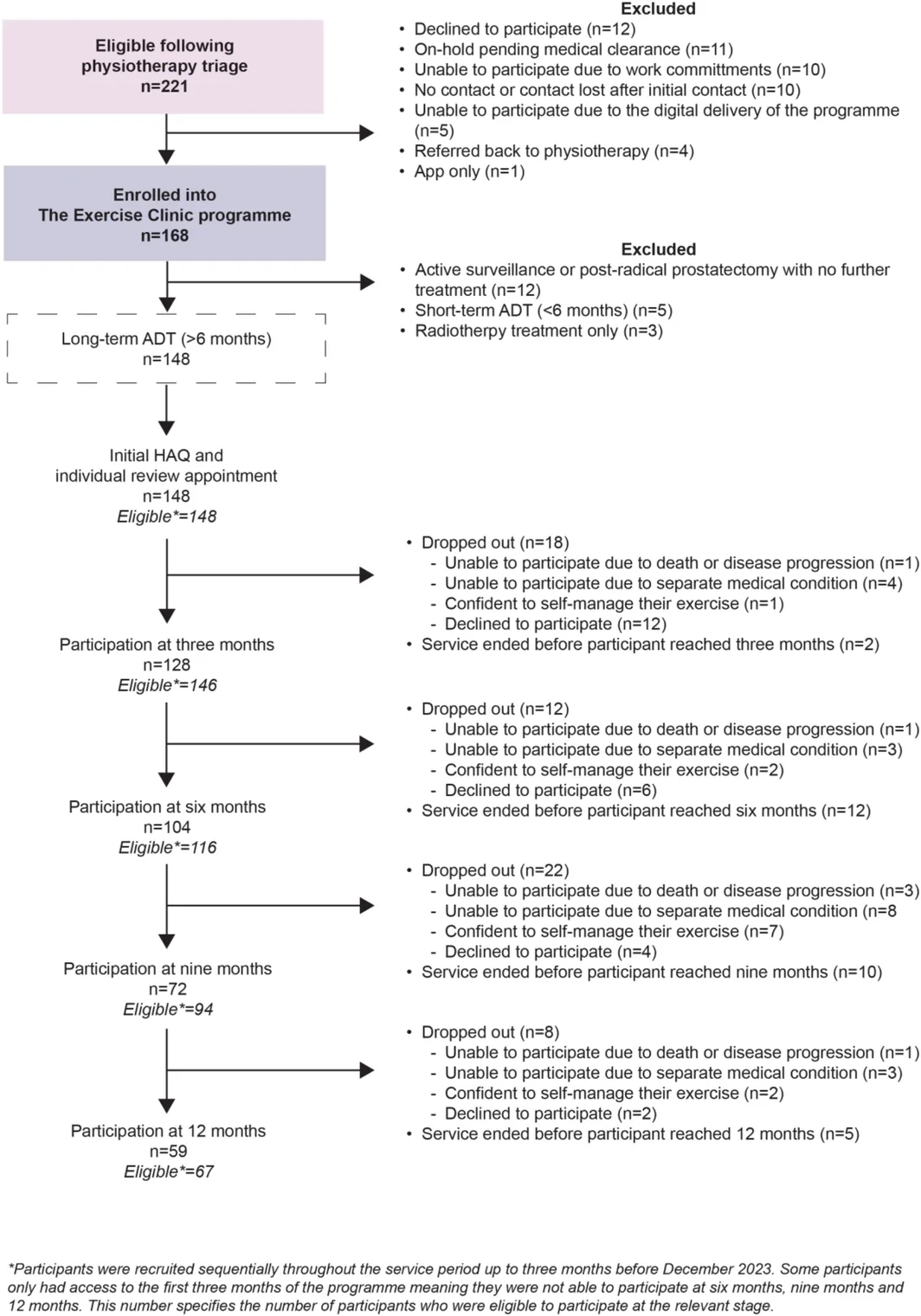
Participant flow diagram.

Participants had a mean (SD) age of 70.6 years (7.8) and BMI of 28.1 (4.4). Eighty‐six (58%) participants had metastatic disease, and, of these, the majority (66 participants) had bone metastases. Sixty‐two (42%) participants had local disease or locally advanced disease and were receiving ADT as a stand‐alone treatment or as an adjunct to radiotherapy. One hundred six (72%) participants reported a side effect of fatigue prior to starting the programme. Full demographic and clinical characteristics are shown in Table S2.

Median duration of participation in TEC was 9 months (IQR 5, 25). Maximum participation duration was 36 months (*n* = 2). One hundred four participants (90% of those eligible) completed their 6‐month review. Fifty‐nine (88% of those eligible) completed their 12‐month review (Figure S1). Participants attended an average of 14 out of 24 scheduled group exercise sessions during their first 6 months in the programme. This includes 19 participants who were only able to attend one session due to work commitments; these participants wanted to participate to access other programme components. TEC app was used by 94 participants (64%), with users submitting an average (median) of 3.6 TEC app reports per month (IQR 2.5, 4.2). Among non‐users (*n* = 54), 36 (67%) had a smartphone but declined use, and 18 (33%) lacked a smartphone. Several patients were advised to contact RM hotline or their oncology team for specialist advice while they were part of TEC but no serious adverse events were reported.

#### 3.1.2 Activity, physical performance and QoL

Average scores for activity levels, physical performance and QoL at each follow‐up point are presented in Table S1. Significant changes were observed in sit‐to‐stand count (mean paired difference = 2.98; 95% CI 5.07, 0.89; *p* = 0.006) and average step count per day (mean paired difference = 638; 95% CI 1313, 52; *p* = 0.035) between quarter one and quarter four. There was a statistically significant change observed in EQ‐5D index value from baseline to 12 months (mean paired difference = 0.07; 95% CI 0.11, −0.03; *p* = 0.002).

#### 3.1.3 Secondary outcomes

Average scores for physical and emotional wellbeing, BMI and waist circumference at each follow‐up timepoint are included in Table S1. No statistically significant changes were seen in BMI and waist circumference from baseline to 12 months. Participants reported higher physical and emotional wellbeing scores at 12 months compared with baseline (mean paired difference = 0.66; 95% CI 1.14, 1.18; *p* = 0.008 and mean paired difference = −0.040; 95% CI 0.73, −0.07; *p* = 0.020, respectively).

### 3.2 Qualitative

#### 3.2.1 Participant satisfaction

During patient discussion groups, 30 participants provided comments about their experience. Framework analysis^27^ comprised four key components: views about group exercise sessions, views about behavioural support, perceived benefits and hopes for the future. Associated data from participants are detailed in Table 1.

**TABLE 1:** Components identified in framework analysis^24^ presented with associated data from participants.

Component		Associated participant data
Views about group exercise sessions^a^	Engaging, convenient, personalised	‘They actively encourage you to improve your fitness […] not by sending you a sheet of exercises to carry out on your own but by providing a weekly face to face zoom meeting with an ever‐changing set of exercises designed to build up your strength and fitness’. P1 ‘Because the group is on Zoom I can dip out of work […] even if I am working from home or at a work venue […] it gives me more opportunities to be flexible and attend when I can’. P17 ‘It is very helpful that the weekly Zoom exercise session is tailored to an individual's abilities and participants are generally in my age group with a similar level of health’. P11
	Motivation and peer support in group setting	‘Whilst it is easy to clock up steps and enjoy the walking activity, working out in the company of others is definitely easier than doing a workout by myself’. P20 ‘The camaraderie of seeing, talking to and taking part in activities with fellow prostate cancer friends is in itself a tonic’. P12
Views about behavioural support^b^	Patient advocate and clinical exercise physiologist	‘The combination of [PA] and [CEP] works well: because [PA] himself is a cancer sufferer we can see the benefits of exercise on managing his illness’. P17 ‘In addition, psychologically knowing a professional is overseeing the workout to help me maximise the exercise effect and watching out for me is very reassuring’. P7
	Motivation for individual exercise	‘Through expert supervision and frequent reviews and updates, I am motivated and very enthusiastic to continue exercising outside the programme’. P23 ‘The sessions help enormously in motivating me, keeping a positive attitude and spurred on to continue exercising on my own’. P5
Perceived benefits of the intervention^c^	Physical benefits	‘[TEC] has been very helpful for my physical improvement, fitness, stamina and strength’. P7 ‘I've lost weight, gained muscle, have great energy levels and I'd bet improved my bone density also’. P15
	Mental health benefits	‘The Group Class […] plus the regular follow up discussions and exercise schedule, has put a spring in my step that has made my life post‐prostate cancer treatment more upbeat, more positive and more fun’. P12 ‘I also feel the Thrive programme has made me more hopeful to deal with the illness going forward’. P14
Hopes for the future^d^	Praise and gratitude	‘The work of the Clinic is literally life‐giving. I feel very fortunate indeed to be able to be part of the programme’. P19 ‘Honestly, The Thrive Project has improved my quality of life […] so much and made me feel different; it has now become one of the most important things in my way of life, it helps me to feel better every day’. P20
	Continue and expand programme	‘I fervently hope that this lifeline continues and is made available more widely to other prostate cancer patients’. P1 ‘Please continue this excellent initiative, which should be open to as many prostate cancer patients as practically possible. In fact, I would suggest that this initiative is copied across the different cancer departments at the Royal Marsden Hospital’. P6

^a^ Participants valued the group sessions for being engaging, with a diversity of exercises; convenient, conducted virtually and at a variety of times during the week; and individualised, according to physical ability and comorbidities. Some participants found the group setting motivational, improving their attendance and effort during sessions. However, the key benefit of the group setting was the opportunity for peer support, which participants found incredibly valuable but not readily available outside TEC.

^b^ Behavioural support provided by both the PC (offering empathetic support) and CEP (providing professional expertise) was noted in half the comments as a particularly effective strategy. This support motivated many participants to engage in independent exercise outside group sessions. Use of TEC app as a means of behavioural support was not commonly raised.

^c^ Ninety per cent of participants praised TEC for improving their physical health, including cardiovascular fitness, flexibility and strength. Equally praised were the mental health benefits, with participants describing renewed optimism and motivation for their cancer journey and wider life.

^d^ Overall, participants expressed overwhelming praise and gratitude for the service. There was a strong desire for it to be continued and expanded to include more patients and other cancer types.

#### 3.2.2 Secondary outcomes

Forty‐nine participants completed the feedback questionnaire (82% response rate) reporting median overall experience of TEC at 10 out of 10 (IQR 9, 10). Self‐reported changes in balance, walking, strength, pain, fatigue, activity levels, wellbeing and exercise confidence are presented in Table 2. For all domains except ‘pain’, the majority of participants reported improvement. The positive effect of the intervention was most notable for domains of ‘strength’, ‘activity levels’, ‘wellbeing’ and ‘exercise confidence’, with over 80% of participants reporting improvements.

**TABLE 2:** Changes in domains of balance, walking, strength, pain, fatigue, activity levels, wellbeing and exercise confidence during participation in The Exercise Clinic service.

Domain^a^	Frequency, *n* (%)^b^		
	Got worse	No change	Improved
Balance	1 (2.1%)	19 (40.4%)	27 (57.5%)
Walking	3 (6.7%)	12 (26.7%)	30 (66.7%)
Strength	0 (0%)	8 (17.4%)	38 (82.6%)
Pain	2 (4.7%)	21 (48.8%)	20 (46.5%)
Fatigue	0 (0%)	11 (24.4%)	34 (75.6%)
Activity levels	1 (2.2%)	5 (10.9%)	40 (87.0%)
Wellbeing	0 (0%)	5 (10.6%)	42 (89.4%)
Exercise confidence	(0%)	9 (19.2%)	38 (80.9%)

^a^ Subjective effectiveness of the intervention on physical, psychosocial and lifestyle domains, assessed via a self‐completed feedback questionnaire.

^b^ Total number of responses for each domain differs as not all respondents gave an answer for each domain.

## 4 DISCUSSION

Referral to THRIVE (266 patients) was lower than anticipated, although the total eligible population and the number of patients who declined referral were unknown. Of the 221 patients eligible for TEC component, 76% agreed to participate. While surveys suggest high willingness among cancer patients to engage in exercise programmes,^28^ actual enrolment in trials is often lower.^29^, ^30^ The comparatively high uptake observed in this service evaluation may reflect the advantages of embedding TEC within routine clinical care^31^ and referral via clinicians,^11^, ^32^ factors previously shown to influence engagement with exercise behaviours in oncology settings.^4^, ^11^, ^12^, ^32^ Fewer than 2% of participants triaged to TEC were re‐referred to physiotherapy, and 5% were placed on hold pending medical clearance, suggesting that physiotherapy‐led assessment was safe and effective in identifying appropriate patients.

Participation and retention in TEC met or exceeded predefined feasibility targets and was consistent with similar supervised exercise interventions.^5^, ^21^, ^33^ While average attendance at group exercise sessions in the first 6 months was lower than reported for supervised programmes,^5^, ^21^ our median includes participants who indicated prior to enrolment that they would not be able to attend more than one session. Notably, engagement with behavioural support components, including quarterly reviews, activity monitoring and individual exercise reports, appeared to facilitate independent PA beyond the supervised setting, suggesting that structured support, including feedback and monitoring, may play an important role in facilitating sustained engagement with exercise. This is promising given uncertainty regarding the sustainability of exercise behaviour following supervised exercise programmes.^9^, ^34^ Participant feedback from other studies has highlighted the importance of structured transitions from supervised exercise to support PA and exercise maintenance in cancer populations.^35^

Participant discussions and self‐reported data indicated improvements in physical, psychological and social wellbeing. Statistically significant changes were observed in selected patient‐reported and functional outcomes over 12 months. However, in the absence of a comparator group, these findings cannot be attributed causally to the intervention. Changes observed may reflect a combination of engagement with the service, participant motivation, or other factors that were not measured. While previous studies suggest that patients receiving ADT typically experience declines in physical function and QoL,^5^, ^31^ this evaluation was not designed to assess effectiveness or mitigation of ADT‐related adverse effects. Findings should therefore be interpreted descriptively.

Participants highlighted the value of combined professional and peer support, suggesting that social interaction and relatable guidance may act as important facilitators of engagement. Peer mentors are shown to be a clinically and cost‐effective adjunct to professional support, improving adherence and outcomes in behavioural interventions across a range of chronic conditions,^36^, ^37^ while individualised exercise prescription delivered by exercise professionals has been shown to be important for achieving therapeutic benefits in cancer patients.^10^ Cormie et al's systematic review concluded that group exercise sessions provide an action‐oriented setting for support that resonates with masculine values of stoicism, self‐sufficiency and independence.^38^ The virtual group exercise environment may offer a unique and accessible form of support and peer interaction, particularly for patients with PCa, who may be less likely to engage with conventional forms of psychological support^5^ and may suffer specific forms of psychosocial distress, such as a perceived loss of masculinity associated with treatment‐related sexual dysfunction.^38^ The contribution of peer support in this context warrants further exploration.

The virtual, home‐based, supervised format of TEC represents a novel hybrid between site‐based supervised exercise and unsupervised home‐based programmes.^12^ Participants cited flexibility and accessibility as major advantages, supporting findings from other digital exercise interventions in PCa care.^39^ While the digital format was acceptable to participants in the service, only those able to engage with the platform were included. Some required additional support to navigate the digital aspects of the intervention, highlighting the need to consider digital literacy and access as factors influencing engagement with digitally delivered interventions. App usage was moderate, with most non‐use attributed to personal preference rather than technological constraints. Nevertheless, digital exclusion remains a concern, particularly for this cohort of older patients. Future research should explore strategies to address this, including patient engagement in programme design and caregiver involvement.^40^

This retrospective evaluation has several limitations. The absence of a control group precludes causal inference regarding effectiveness. Outcomes were largely self‐reported and subject to reporting and desirability bias. Participants were recruited sequentially over time and varied in disease stage and treatment, limiting our ability to draw conclusions about specific clinical sub‐groups. In addition, the delivery and evaluation of the service involved staff employed by the service provider, which may have influenced participant feedback despite efforts to ensure that reporting was transparent.

The referral and triage procedure meant participants were well‐functioning individuals with motivation, physical capability and technological ability to undertake a virtual exercise programme. Data from patients excluded or who declined to participate were limited, introducing selection bias and restricting generalisability. Consequently, our findings may not reflect the experiences or outcomes of patients receiving ADT for PCa, particularly those with more complex multimorbidity or with limited access to digital technologies and should therefore be interpreted cautiously.

This service evaluation demonstrates that a virtually delivered, supervised group exercise programme embedded within NHS care is feasible and acceptable for a selected group of men receiving long‐term ADT. High levels of engagement and satisfaction, alongside encouraging descriptive outcomes, support further investigation of digitally delivered exercise services in PCa survivorship. Future research should prioritise randomised controlled trials to assess clinical effectiveness and cost‐effectiveness, alongside qualitative work to better understand factors influencing engagement and to ensure interventions are inclusive, scalable and aligned with patient needs.

## AUTHOR CONTRIBUTIONS

Emily Curtis, Chris Cottrell, Siobhan Cowan‐Dickie and Alison Reid contributed to the conception and design of the study. Emily Curtis contributed to study design, data collection, data analysis, interpretation of results and writing the manuscript. Author Alice Russell contributed to data analysis, interpretation of the data and the initial draft of the manuscript. Author Chris Cottrell contributed to study design, data collection, data analysis, interpretation of results and writing the manuscript. Authors Siobhan Cowan‐Dickie, Sarah Dewhurst and Alison Reid provided clinical supervision, contributed to interpretation of the findings and critically revised the manuscript. All authors reviewed and approved the manuscript.

## CONFLICT OF INTEREST STATEMENT

Emily Curtis and Chris Cottrell hold shares in The Exercise Clinic Ltd and delivered TEC programme as the clinical exercise physiologist and patient contributor, respectively. The authors have no other conflicts of interest to declare.

## Supporting information


**Figure S1.** Participant engagement and dropout at each stage.
**Table S1.** Changes in QoL, wellbeing, activity levels, physical performance, BMI and waist circumference during participation in The Exercise Clinic service.
**Table S2.** Baseline characteristics of participants on long‐term ADT (for a period of more than six months) enrolled in The Exercise Clinic service (*n* = 148).
