# Supplementary material for: Participation and satisfaction of prostate cancer patients on androgen deprivation therapy participating in a virtual supervised group exercise service
Source: BJUI Compass. 2026 Sep 30;7(10):e70243. doi: 10.1002/bco2.70243 (PMC13624836; doi:10.1002/bco2.70243)
Supplement: Supplementary file 1 [file BCO2-7-e70243-s001.docx]

**Figure S1.** Participant engagement and dropout at each stage

**Table S1.** Changes in QoL, wellbeing, activity levels, physical performance, BMI and waist circumference during participation in The Exercise Clinic service.

| **Outcome measure** | **Mean (SD) value**^a^ | | | | | **Change from baseline to 12 months**^a^ | | | |
| --- | --- | --- | --- | --- | --- | --- | --- | --- | --- |
|  | **Baseline** | **3 months** | **6 months** | **9 months** | **12 months** | **Mean difference**^b^ | **Df** | **95% CI** | **p-value**^c^ |
| **EQ-5D index value**^d^ | 0.72 (0.14) | 0.75 (0.13) | 0.76 (0.12) | 0.77 (0.13) | 0.77 (0.11) | 0.07 | 45 | -0.03 to 0.11 | 0.002 |
| **Emotional wellbeing** | 7.2 (1.7) | 7.3 (1.5) | 7.3 (1.7) | 7.3 (1.6) | 7.7 (1.4) | 0.40 | 53 | -0.07 to 0.73 | 0.020 |
| **Physical wellbeing** | 6.2 (1.8) | 6.7 (1.6) | 6.9 (1.5) | 7.0 (1.4) | 7.2 (1.3) | 0.66 | 53 | 0.18 to 1.14 | 0.008 |
| **Godin** **score**^e^ | 33.7 (26.3) | 46.8 (34.5) | 40.5 (22.2) | 43.6 (30.8) | 39.2 (23.7) | 4.02 | 53 | -3.17 to 11.20 | 0.267 |
| **Step count**, *steps per day* | ^f^ | 5775 (3056) | 6045 (2854) | 5396 (2562) | 6027 (3224) | 683^g^ | 38 | 52 to 1313 | 0.035 |
| **Sit-to-Stand** | ^f^ | 31.7 (10.9) | 34.6 (10.5) | 35.8 (11.4) | 36.2 (11.4) | 2.98^g^ | 44 | 0.89 to 5.07 | 0.006 |
| **BMI**, *kg/m^2^* | 28.1 (4.4) | 27.9 (4.5) | 27.9 (5.0) | 27.6 (5.2) | 27.0 (4.6) | 0.11 | 52 | -0.18 to 0.40 | 0.439 |
| **Waist circumference**, *cm* | 96.4 (9.1) | 97.7 (9.3) | 96.3 (8.9) | 95.4 (9.8) | 95.9 (8.4) | 0.78 | 51 | -0.07 to 2.30 | 0.310 |
| a. *Mean (SD) values include data on all participants who were eligible to participate at that stage. Data for participants who had not reached 12 months or had dropped out before this point is not included in the Change from baseline to 12 months.* b. *Mean paired difference calculated as 12-month value minus baseline value.* c. *Paired t-test.* d. *EQ-5D index value as a measure of quality of life; higher scores represent better self-reported quality of life.* e. *Godin score as a measure of physical activity; higher scores represent a higher level of physical activity.* f. *Data not collected at baseline.* g. *Mean paired difference calculated as 12-month value minus 3-month value.*   *Abbreviations: BMI, body mass index; SD, standard deviation; Df, degrees of freedom (number of observations-1); CI, confidence interval.* | | | | | | | | | |

**Table S2.** Baseline characteristics of participants on long-term ADT (for a period of more than six months) enrolled in The Exercise Clinic service (n=148).

| **Baseline characteristic** | **Results presented as mean ± SD, median (IQR), or number of participants (percentage)** |
| --- | --- |
| **Age**, *years* | 70.6 (7.8) |
| **Weight**, *kg* | 86.5 (12.7) |
| **Height**, *cm* | 175.6 (7.6) |
| **BMI**, *kg/m^2^* | 28.1 (4.4) |
| **Prevalence or more comorbidities**^a^ | 89 (60%) |
| **Side effect of fatigue** | 106 (72%) |
| **Hypertensive** | 91 (61%) |
| **Tumour grade** | |
| Local | 46 (31%) |
| Locally advanced^b^ | 16 (11%) |
| Metastatic | 86 (58%) |
| **Indication for ADT** | |
| Adjuvant | 46 (31%) |
| Biochemical relapse | 8 (5%) |
| Metastases | 94 (64%) |
| **Clinical history**^c^ | |
| Previous prostatectomy | 17 (11%) |
| Previous or concurrent radiotherapy^d^ | 110 (74%) |
| Previous or concurrent chemotherapy^e^ | 22 (15%) |
| Concurrent ARPI^f^ | 91 (61%) |
| a. *Cardiovascular disease, diabetes, respiratory disease, liver disease, renal disease, neurological disease, arthritis, osteoporosis, other primary malignancy, mental health condition.* b. *Of these participants, some (n=8) were classified as locally advanced with negligible lymph node involvement. These participants are included in Indication for ADT Metastases group but not the Tumour grade Metastatic group.* c. *Some participants may fulfil more than one Clinical history category.* d. *Previous radiotherapy or receiving radiotherapy concurrent to participation in TEC.* e. *Previous chemotherapy or receiving chemotherapy concurrent to participation in TEC.* f. *Receiving ADT in conjunction with abiraterone, enzalutamide, apalutamide or darolutamide.* | |
